# Danger of Pregnancy Following Operations for Cancer of the Breast1Read at the fortieth annual meeting of the Medical Association of Central New York, held at Rochester, October 15, 1907.

**Published:** 1908-01

**Authors:** William S. Cheesman

**Affiliations:** Auburn, N. Y.


### Danger of Pregnancy Following Operations for Cancer of the Breast[1]

By WILLIAM S. CHEESMAN, Auburn, N. Y.

WHATEVER theory we adopt as to the nature and etiology of cancer in general, it must be conceded that when located in the female breast its development is influenced by some unexplained sympathetic correlation with the pelvic organs. The clinical fact has long been recognised, and is sometimes mentioned in textbooks, that under the physiological stimulus of pregnancy mammary cancer takes on a specially malignant character. And on the other hand, Beatson by ablating the ovaries in some cases of late inoperable cancer of the breast, was able to effect the disappearance of the disease. So we may say of this mysterious epithelial reproduction, this cellular new birth, to which we give the name cancer, that whatever its ultimate character, it may be stimulated to unwonted efflorescence, or retarded and even extinguished, according as the uterus and appendages are rendered active or functionally obsolete.

The highest functional act of these organs is gestation. This may associate itself with mammary cancer in one of two ways; either cancer attacks the breast during the course of pregnancy, or pregnancy occurs as a complication of already existing cancer. In which ever way the association arranges itself the result

---

1. Read at the fortieth annual meeting of the Medical Association of Central New York, held at Rochester, October 15, 1907.

is the same, viz.: a stimulation of the disease to unexampled malignancy and rapidity of growth.

It has been my evil fortune to encounter each of these two varieties.

CASE 1.—*Cancer of the Breast Complicating Pregnancy.*—About a dozen years ago a lady aged 29, mother of three children, became pregnant for the fourth time. She had had an abscess of the breast after her first confinement, and there remained a nearly imperceptable cicatrix in the gland. This was quiescent during two succeeding pregnancies, but about the third month of her fourth pregnancy she reported that the old scar was enlarged and tender. Examination showed a swollen, indurated mass in the breast, and axillary and supra-clavicular glands enlarged. A well known surgeon saw her at once with me, diagnosticated malignancy of rapidly growing type, and did a thorough extirpation of breast and all lymphatic connections. The wound healed kindly, but in spite of the sweeping thoroughness of removal the disease seemed scarcely to have been checked. It broke out immediatly over the whole area of operation, ulcerating and discharging and finally involving the pleura. I induced labor early in the eighth month, saving the child, now a well grown boy; but the young mother died a few weeks later.

This case familiarised me with the behavior of cancer of the breast during gestation, but it needed still another to awaken my dull perceptions to the importance of this knowledge for the surgeon.

CASE 2.—*Pregnancy Complicating Cancer of the Breast.*—In March, 1905, I operated on a woman aged 36, doing the usual removal of breast, axillary contents, and muscles, by a wide circumsection necessitating Thiersch skin-grafts to close the defect. I mention these details to show that the work was thorough. The wound healed well, only a soft pliable scar remaining. I watched this case from time to time, and more and more felt that the result promised at least long postponement of return. All went smoothly till in December, 1905, (nine months after operation) the patient reported herself pregnant two months, and examination verified this suspicion. Her danger was instantly clear to me, and I told her the pregnancy should be interrupted in order to avert the chance of its relighting the disease. This view being concurred in by a consultant, I emptied the uterus of a two months' embryo.

But even at the second month we were too late. I had observed the scar to be a trifle red and indurated just before the curettage, but soon after there could be no doubt. A flame of reddened lymphatics spread from the scar to the other breast which was swollen and glossy with indurated edema. ("Mastitis carcinomatosa" of Volkmann). The situation was so dreadfully clear that the patient herself recognised it, and asked: "Did my pregnancy bring this back again?" I had no need to answer; she read the truth, and pierced my conscience with the searching query: "Then, why did you not warn me?"

I have asked myself that question many times since, and I would ask my colleagues today: *Why have we not warned breast cases of the dangers of pregnancy?* Of course the cases suffering from our omission have been few in number, unfortunate rarities, I judge, of surgical experience. Cancer attacks the breast commonly late in life, well after the child-bearing period. But not exclusively then. The circles of incidence of the two conditions intersect and overlap to a considerable extent. So that there is a period of ten to fifteen years in which a small percentage of women are liable to both conditions. But even if the number of such women were too small to affect the statistics underlying operative prognosis, even if we see but one or two such cases in a long surgical experience, we shall not escape the condemnation of conscience if we fail to individualise in their favor, and admonish them of their peril.

I do not know whether others have recognised this danger and this duty to their patients. If so, they have failed to indicate it in the literature. A research carried on for me in the library of the Syracuse Academy of Medicine fails to bring to light any evidence that the subject has received attention. I am constrained, therefore, to believe that the danger has not been clearly appreciated, and that once notice is drawn to it, others will wish, like myself, to include in the advice given patients after operations for malignancy of the breast, a warning against pregnancy.

